# Cardioprotective Effect of *Croton macrostachyus* Stem Bark Extract and Solvent Fractions on Cyclophosphamide-Induced Cardiotoxicity in Rats

**DOI:** 10.1155/2020/8467406

**Published:** 2020-03-31

**Authors:** Muluken Altaye Ayza, Rajkapoor Balasubramanian, Abera Hadgu Berhe

**Affiliations:** ^1^Department of Pharmacology and Toxicology, School of Pharmacy, Mekelle University, Mekelle, Ethiopia; ^2^Department of Pharmacology, JKK Nattraja College of Pharmacy, Komarapalayam-638 183, Namakkal Dist, Tamilnadu, India

## Abstract

**Objective:**

To evaluate the antioxidant and cardioprotective activities of stem bark extract and solvent fractions of *Croton macrostachyus* on cyclophosphamide-induced cardiotoxicity in rats. *Materials and Methods*. DPPH free radical scavenging assay method was used to determine antioxidant activity whereas Sprague-Dawley rats were used to evaluate the cardioprotective activity. Except for the normal control, all groups were subjected to cyclophosphamide (200 mg/kg, i.p.) toxicity on the first day. Enalapril at 10 mg/kg was used as a reference. The hydromethanolic crude extract (100, 200, and 400 mg/kg) and aqueous and ethyl acetate fractions (100 and 200 mg/kg, each) were administered for 10 days. The cardioprotective activities were evaluated using cardiac biomarkers such as Troponin I, aspartate aminotransferase (AST), alanine aminotransferase (ALT), alkaline phosphatase (ALP), total cholesterol (TC), triglyceride (TG), and histopathological studies of heart tissue.

**Results:**

Crude extract and ethyl acetate and aqueous fractions exhibited free radical scavenging activities at IC_50_ of 594 *μ*g/mL, 419 *μ*g/mL, and 716 *μ*g/mL, respectively. Crude extract at 400 mg/kg decreased the levels of troponin, AST, ALT, and ALP to 0.29 ± 0.06 ng/mL, 103.00 ± 7.63 U/L, 99.80 ± 6.18 U/L, and 108.80 ± 8.81 U/L, respectively. In addition, ethyl acetate fraction at 200 mg/kg decreased the levels of troponin, AST, ALT, and ALP to 0.22 ± 0.02 ng/mL, 137.00 ± 14.30 U/L, 90.33 ± 6.13 U/L, and 166.67 ± 13.50 U/L, respectively, compared with the cyclophosphamide control group.

**Conclusions:**

*Croton macrostachyus* possesses cardioprotective activities and it could be a possible source of treatment for cardiotoxicity induced by cyclophosphamide.

## 1. Introduction

Cardiotoxicity induced by drugs poses a serious risk to human health and is becoming an increasingly important concern in cardiooncology [1]. Improvements in antineoplastic treatments led to increased overall and progression-free survival in the management of an increasing number of malignancies [2]. However, as cancer survival has improved with advancing therapy, late cardiovascular side effects have become an important management issue, particularly in childhood cancers, lymphoma, leukemia, and breast cancer [3].

Cancer survivors, when compared with their healthy counterparts, are at high risk of cardiovascular-related deaths, including myocardial infarction with coronary artery disease, cardiomyopathy with congestive heart failure, and cerebrovascular events [4, 5]. People on cardiotoxic chemotherapy can be considered as a stage A group of heart failure patients [6].

Anticancer drugs are well known to cause a wide array of toxicities, including cardiac damage; these may include cardiac dysfunction leading to heart failure, myocardial ischemia, arrhythmias, hypertension, myocarditis, pericarditis, and thromboembolism [7]. Alkylating drugs, including cisplatin, cyclophosphamide, ifosfamide, carmustine, chlormethine, busulfan, and mitomycin are especially associated with cardiac toxicity [8].

Mechanisms of cyclophosphamide-induced cardiotoxicity encompass oxidative and nitrative stress, protein adduct formation which leads to cardiomyocyte inflammation, altered calcium homeostasis, programmed cell death, swelling of the cardiomyocytes, nuclear splitting, vacuolization, and alteration in signaling pathways. These events result in diseases of the heart muscle including heart failure, and if left untreated, this may result in death [9].

Cyclophosphamide-induced cardiac damage is dose-dependent and the total dose of an individual course is the best indicator of toxicity; patients who receive greater than 150 mg/kg or 1.55 g/m2/day are at high risk for cardiotoxicity [10]. Cardiotoxicity is a dose-limiting factor during cyclophosphamide therapy [11]. Although fatal cardiomyopathy has been reported among 2–17% of patients, it depends on the regimen and the patient population [12].

Various natural antioxidants originated from medicinal plants, which are used for the treatment of different ailments throughout the world. *Croton macrostachyus* Hochst. ex Delile (Euphorbiaceae) is mostly known as rush foil or wide-leaved croton in English. It is a deciduous tree of the family Euphorbiaceae, a very large family with 300 genera and 8,000 to 10,000 species. Eight of these species (*Croton dichogamus* Pax, *Croton zambesicus* Muell. Arg, *Croton menyhartii* Pax, *Croton somalense*, *Croton schimperianus* Müll. Arg, *Croton sylvaticus* Hochst. ex Krauss, *Croton lobatus* Linn., and *Croton macrostachyus* Hochst. ex Delile) are found in Ethiopia [13].

Traditional healers use *Croton macrostachyus* to treat various human diseases [14]. Some of these ethnomedicinal uses of *Croton macrostachyus* were validated in several experimental studies. The plant showed antibacterial [15], antidiarrheal [16], anticonvulsant [17], antidiabetic [14], and antihyperalgesic activity [18].

The phytochemical screening showed that the stem bark of *Croton macrostachyus* constituted the major secondary metabolites such as tannins, steroids, alkaloids, phenols, terpenoids, saponins, and flavonoids, which might be the reason for the plant widespread pharmacological activity [19, 20].

Though *Croton macrostachyus* Hochst. ex Delile has been used in Ethiopian traditional medicine to treat different ailments including heart diseases, scientific investigation on pharmacological actions related to the cardioprotective activity has not been carried out. Therefore, the present study investigates an *in vitro* antioxidant activity and *in vivo* cardioprotective effects of *Croton macrostachyus* on cyclophosphamide-induced cardiotoxicity in rats.

## 2. Materials and Methods

### 2.1. Chemicals, Reagents, and Drugs

Cyclophosphamide injection IP (Cadila Healthcare Limited, India), ketamine hydrochloride (Neon Laboratories Limited, India), 2, 2-diphenyl-1-picrylhydrazyl (DPPH) (Tokyo Chemical Industry Co., Ltd., Japan), methanol (Carlo Erba Reagents S.A.S), ethyl acetate, n-hexane (Loba Chemie PVT. Ltd.), formalin 10% (Sheba Pharmaceuticals PLC, Ethiopia), normal saline (Addis Pharmaceutical Factory, Ethiopia), and distilled water (Jourilabs, Ethiopia) were used. All other chemicals used were also of analytical grade.

### 2.2. Collection of Plant Material

Fresh *Croton macrostachyus* stem bark was collected on 25/11/2018 from Abiy-Adi, Tigray, Ethiopia, 101.5 km away from the regional capital Mekelle, and 1,031 km towards North from Addis Ababa, Ethiopia. Identification and authentication of the plant were carried out at the Department of Plant Biology and Biodiversity Management, Addis Ababa University, and sample specimen was deposited at the National Herbarium of Ethiopia with Ref. No. ETH/4/2011/2019.

### 2.3. Preparation of Crude Extract and Solvent Fractions

The collected fresh stem bark was washed in order to remove dead materials and dust and then allowed to dry for three weeks under a shade. The dried stem bark was then pulverized, using a grinder. The powder (1.52 kg) was then macerated in 80% methanol for 72 h in maceration jars; extraction was aided by an orbital shaker and intermittent stirring.

For exhaustive extraction of the plant material, the residue was remacerated for another 72 h twice. The extract was then filtered using a muslin cloth followed by Whatman filter paper No. 1. The combined filtrates were dried in an oven at a temperature of 40°C. Then, the dried extract (crude extract) was weighed and kept in a tightly closed amber bottle and stored in a refrigerator at 4°C until further use.

Fractionation was carried out using a separatory funnel. Sixty-five grams of the crude extract was dissolved in 325 mL of distilled water. Then, the extraction was performed by using 325 mL hexane, for three consecutive times, followed by 325 mL ethyl acetate again three times in order to attain complete fractionation. After collecting the hexane and ethyl acetate fractions, the remaining residue was considered as an aqueous fraction. The fractions were concentrated using an oven dryer at 40°C and weighed. Hexane fraction was yellowish oily. Dried powders of the aqueous and ethyl acetate fractions were kept in an airtight container and stored in a refrigerator at 4°C until further use.

### 2.4. Experimental Animals

Female Swiss albino mice aged 8–12 weeks were used for the acute toxicity study. For the main study, either sex of Sprague-Dawley (SD) rats (3–4 months ages) was obtained from the Department of Pharmacology and Toxicology, School of Pharmacy, Mekelle University. Animals were housed in a room with a 12 h light/dark cycles and provided with standard pellet feed and water *ad libitum*.

The animals were acclimatized to the experimental environment prior to use for the study. All the experiments were conducted in accordance with the international laboratory animal use and care guideline. The study clearance was obtained from the Health Research Ethics Review Committee (HRERC) of the College of Health Sciences, Mekelle University with protocol number 1536/2018.

### 2.5. Acute Oral Toxicity Test

The acute oral toxicity test was carried out according to the Organization for Economic Cooperation and Development Guideline No. 425 [21]. Five healthy, nulliparous, nonpregnant female Swiss albino mice (aged 8–12 weeks) weighing 25–30 g were used for this test. The mice were fasted for food but not water 4 h prior to dosing and 1 h after the administration of the extract.

A single dose of the extract (2000 mg/kg) was administered using oral gavage to the first mouse which was subsequently observed for any physical and behavioral changes. After 24 hours, 2000 mg/kg single dose was administered orally to each of the remaining four mice. The mice were then observed individually for gross behavioral changes (locomotion, activity, hair texture, pupil size, and feeding) at least once during the first 30 min, periodically for 24 h, with special attention given during the first 4 h, and daily observation were made thereafter for a total period of 14 days.

### 2.6. Determination of Antioxidant Activity (DPPH) Assay

To determine the free radical scavenging activity of the crude extract and solvent fractions, the *in vitro* DPPH assay method was used [22]. 3 mL of 0.004% DPPH in methanol was mixed with 1 mL of different concentrations (200, 100, 50, 25, and 12.5 *μ*g/mL) of crude and solvent fraction of *Croton macrostachyus* and ascorbic acid (a reference compound) separately. The mixture was vortexed and left for half hour at room temperature in the dark for incubation, and then, the absorbance of the mixture in the samples was measured using a spectrophotometer at 517 nm against methanol as blank. Lower absorbance values of reaction mixture designate higher free radical scavenging activity. Percentage radical scavenging activities of the test samples were calculated by comparison with a control (3 mL DPPH solution mixed with 1 mL methanol). All samples were measured in triplicate and the average was calculated.

The percentage of radical scavenging activity (RSA) was calculated using the following formula:

(1) $$\begin{array}{c} \%\text{RSA}=\left[\frac{\left(A0-A1\right)}{A0}\right]\times 100, \end{array}$$

where *A*0 is the absorbance of the control, and *A*1 is the absorbance of samples.

The free radical scavenging activity of the extracts and ascorbic acid was expressed as a half-maximal inhibitory concentration (IC50). The IC50 value is defined as concentration (in *μ*g/mL) of the sample that inhibits 50% of the DPPH radical.

### 2.7. Grouping and Dosing of Animals

To evaluate the cardioprotective activity, cyclophosphamide-induced cardiotoxicity damage method was used, as described by Mythili et al. [23], Viswanatha Swamy et al. [24], Alhumaidha et al. [25], Bhatt et al. [26], and Ogunsanwo et al. [27]. The rats were divided into thirteen groups (Table 1); each group consist of six animals.

For the crude extract and aqueous fractions, group I served as normal control (NC) (received normal saline orally for 10 days), group II served as cyclophosphamide (CP) control (received a single dose of CP at 200 mg/kg, i.p, dissolved in normal saline on the first day of the experiment period), group III served as standard control (received CP at 200 mg/kg, i.p, on the first day and then enalapril at 10 mg/kg orally for 10 days), and groups IV-VIII were treatment groups (received CP at 200 mg/kg, i.p, on the first day and then three doses of crude extract, 100, 200, and 400 mg/kg, and 100 and 200 mg/kg of the aqueous fraction dissolved in normal saline orally for 10 days, respectively). For the ethyl acetate fraction, group IX served as normal control (NC) (received 2% dimethyl sulfoxide (DMSO) orally for 10 days), group X served as CP control (received a single dose of CP at 200 mg/kg, i.p, on the first day of the experiment period), group XI served as standard control (received CP at 200 mg/kg, i.p, on the first day and then enalapril 10 mg/kg orally for 10 days), and groups XII and XIII served as treatment group (received CP at 200 mg/kg, i.p, on the first day and then 100 and 200 mg/kg of the ethyl acetate fraction dissolved in 2% dimethyl sulfoxide orally for 10 days, respectively).

### 2.8. Blood Collection

After 24 h of the last dose, all rats were anesthetized under ketamine anesthesia (75 mg/kg, i.p) and blood was collected by the retroorbital puncture using microcapillary tubes. Then, the serum was separated through centrifugation at 5000 rpm for 10 min and used for the estimation of marker enzymes.

### 2.9. Determination of Cardiac Markers

The separated serum was used for the estimation of marker enzymes. Troponin I was determined using CS-200 acute care analyzer whereas aspartate aminotransferase (AST), alanine aminotransferase (ALT), alkaline phosphatase (ALP), total cholesterol (TC), and triglyceride (TG) levels were measured using BTS 350 semiautomated biochemistry analyzer.

### 2.10. Histopathological Examination

The heart was immediately removed from animals and washed with water and blotted dry with filter paper and weighed and then fixed in 10% formalin for histopathological analysis. Histological sections of the heart were stained with hematoxylin and eosin (H&E). Heart sections were observed for myocyte damage, multifocal degeneration, inflammation, myofibrillar degeneration, and necrosis.

### 2.11. Statistical Analysis

The data were expressed as means ± standard error of the mean (SEM). The analysis was carried out using the statistical package for social science (SPSS version 20.0). One-way analysis of variance (ANOVA) followed by Tukey's post hoc test was applied to compare variations among groups. *P* < 0.05 was considered statistically significant.

## 3. Results

### 3.1. Extraction Yields of Plant Material

The percentage yield of crude extract was 6.68%. Among the fractions, the aqueous fraction was the one with the highest yield at 56.46%. The ethyl acetate fraction was 17.53%.

### 3.2. Acute Oral Toxicity Test

Administration of 2000 mg/kg of the *Croton macrostachyus* did not produce any sign of toxicity. It did not cause changes in breathing, alertness, and motor activity. There were no restlessness, diarrhea, coma, and convulsions in the two-week follow-up period. Therefore, the LD50 of the crude extract can be considered to be higher than 2000 mg/kg.

### 3.3. DPPH Free Radical Scavenging Activity

The ethyl acetate fraction exhibited the highest free radical scavenging activity with IC_50_ of 419 *μ*g/mL when compared with crude extract and aqueous fraction (IC_50_ of 594 *μ*g/mL and 716 *μ*g/mL, respectively). The IC_50_ of standard solution (ascorbic acid) was 30.612 *μ*g/mL with *R*^2^ = 0.975.

### 3.4. Effect of Crude Extract and Solvent Fractions on Body Weight

As shown in Table 2, significant weight loss was observed in the cyclophosphamide-treated group when compared with a normal control group (*P* < 0.001). However, the treatments with 100 mg/kg crude extract and 10 mg/kg enalapril significantly prevented weight loss compared with the group treated with cyclophosphamide alone (*P* < 0.05 and *P* < 0.01). Treatment with aqueous fractions also significantly prevented the weight loss compared with the cyclophosphamide control group (*P* < 0.001) (Table 2).

In the ethyl acetate fraction group, the administration of cyclophosphamide resulted in significant weight loss when compared with the normal group animals (*P* < 0.001). Treatment with ethyl acetate fraction (100 and 200 mg/kg) and enalapril (10 mg/kg) prevented weight loss significantly when compared with the cyclophosphamide control group (*P* < 0.001) (Table 3).

### 3.5. Effect of Crude Extract and Solvent Fractions on Heart Weight and Heart-to-Body Weight Ratio

Cyclophosphamide significantly (*P* < 0.001) increased heart weight as well as the heart-to-body weight ratio compared with the normal control group (Table 2). 400 mg/kg crude extract and 10 mg/kg enalapril treatment significantly prevented heart weight increment when compared with cyclophosphamide-treated group (*P* < 0.001). Moreover, treatment with crude extract at 100 and 200 mg/kg significantly prevented heart weight increment compared with cyclophosphamide-treated group (*P* < 0.01).

Similarly, treatment with enalapril at 10 mg/kg and aqueous and ethyl acetate fractions at 100 and 200 mg/kg decreased heart-to-body weight ratio significantly compared with the cyclophosphamide control group (*P* < 0.001) (Table 3).

### 3.6. Effect of Crude Extract and Solvent Fractions on Cardiac Biomarkers

**Figure 1:**
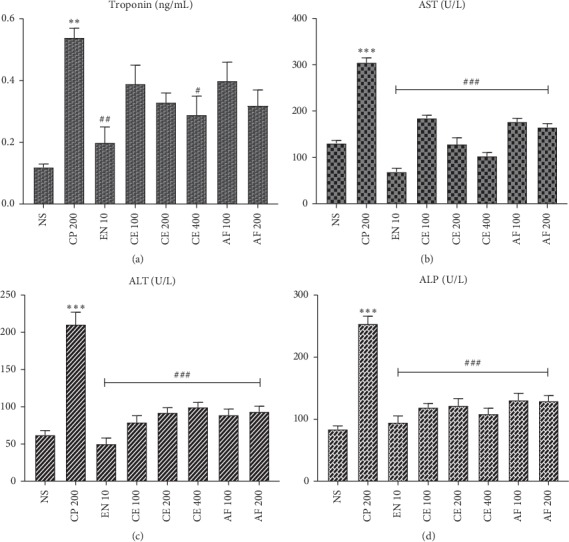
Effect of crude extract and aqueous fraction of *Croton macrostachyus* on the serum levels of (a) troponin, (b) aspartate aminotransferase (AST), (c) alanine aminotransferase (ALT), and (d) alkaline phosphatase (ALP). Results are expressed as mean ± SEM (*n* = 6). ^*∗∗*^*P* < 0.01, ^*∗∗∗*^*P* < 0.001 when compared wih normal control; ^#^*P* < 0.05, ^##^*P* < 0.01, and ^###^*P* < 0.001 when compared with CP control. AF: aqueous fraction; CP: cyclophosphamide; CE: crude extract; EN: enalapril; NS: normal saline.

Serum cardiac biomarkers such as troponin (*P* < 0.01) and AST, ALT, and ALP (*P* < 0.001) were significantly increased by the administration of cyclophosphamide compared with normal control animals (Figure 1). Treatment with crude extract and aqueous fraction significantly decreased the levels of AST, ALT, and ALP compared with the cyclophosphamide-treated group (*P* < 0.001). The crude extract (400 mg/kg) decreased troponin level significantly compared with the cyclophosphamide-treated group (*P* < 0.05).

**Figure 2:**
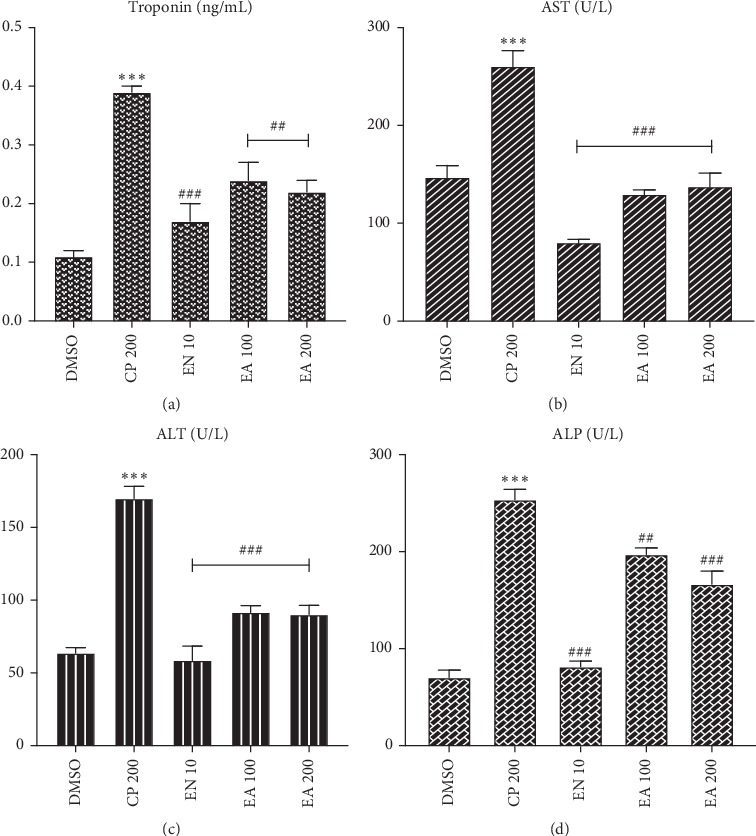
Effect of ethyl acetate fraction of *Croton macrostachyus* on the serum levels of (a) troponin, (b) aspartate aminotransferase (AST), (c) alanine aminotransferase (ALT), and (d) alkaline phosphatase (ALP). Results are expressed as mean ± SEM (*n* = 6). ^*∗∗∗*^*P* < 0.001 when compared with normal control; ^#^*P* < 0.05, ^##^*P* < 0.01, and ^###^*P* < 0.001 when compared with CP control. DMSO: dimethyl sulfoxide; EA: ethyl acetate fraction; CP cyclophosphamide; CE: crude extract; EN: enalapril.

Likewise, treatment with ethyl acetate fraction (100 and 200 mg/kg) significantly decreased serum cardiac biomarkers compared with cyclophosphamide. Ethyl acetate fraction (100 and 200 mg/kg) significantly reduced the troponin level; the reduction is comparable to enalapril-treated animals (Figure 2).

### 3.7. Effect of Crude Extract and Solvent Fractions on Lipid Profile

**Figure 3:**
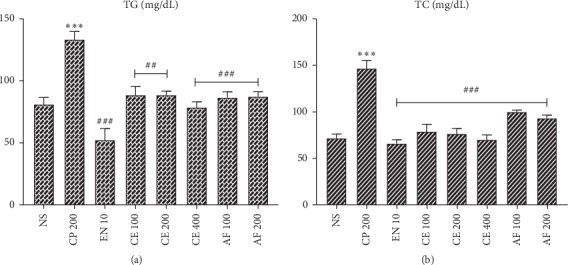
Effect of crude extract and aqueous fraction of *Croton macrostachyus* on the serum levels of (a) triglyceride (TG) and (b) total cholesterol (TC). Results are expressed as mean ± SEM (*n* = 6). ^*∗∗∗*^*P* < 0.001 when compared with normal control; ^##^*P* < 0.01 and ^###^*P* < 0.001 when compared with CP control. AF: aqueous fraction; CP: cyclophosphamide; CE: crude extract; EN: enalapril; NS: normal saline.

**Figure 4:**
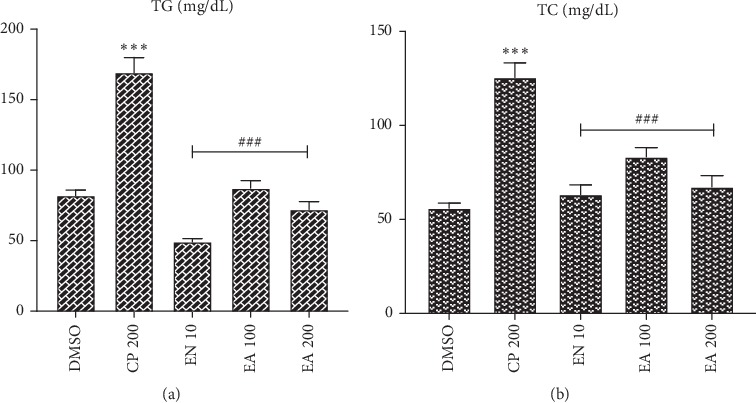
Effect of ethyl acetate fraction of *Croton macrostachyus* on the serum levels of (a) triglyceride (TG) and (b) total cholesterol (TC). Results are expressed as mean ± SEM (*n* = 6). ^*∗∗∗*^*P* < 0.001 when compared with normal control; ^#^*P* < 0.05, ^##^*P* < 0.01, and ^###^*P* < 0.001 when compared with CP control. DMSO: dimethyl sulfoxide; EA: ethyl acetate fraction; CP: cyclophosphamide; CE: crude extract; EN: enalapril.

Cyclophosphamide administration significantly increased both TG and TC levels compared with normal control group of animals (*P* < 0.001). All treatment groups significantly decreased both TG and TC levels when compared with cyclophosphamide-treated group of animals (Figures 3 and 4).

### 3.8. Histopathological Studies

**Figure 5:**
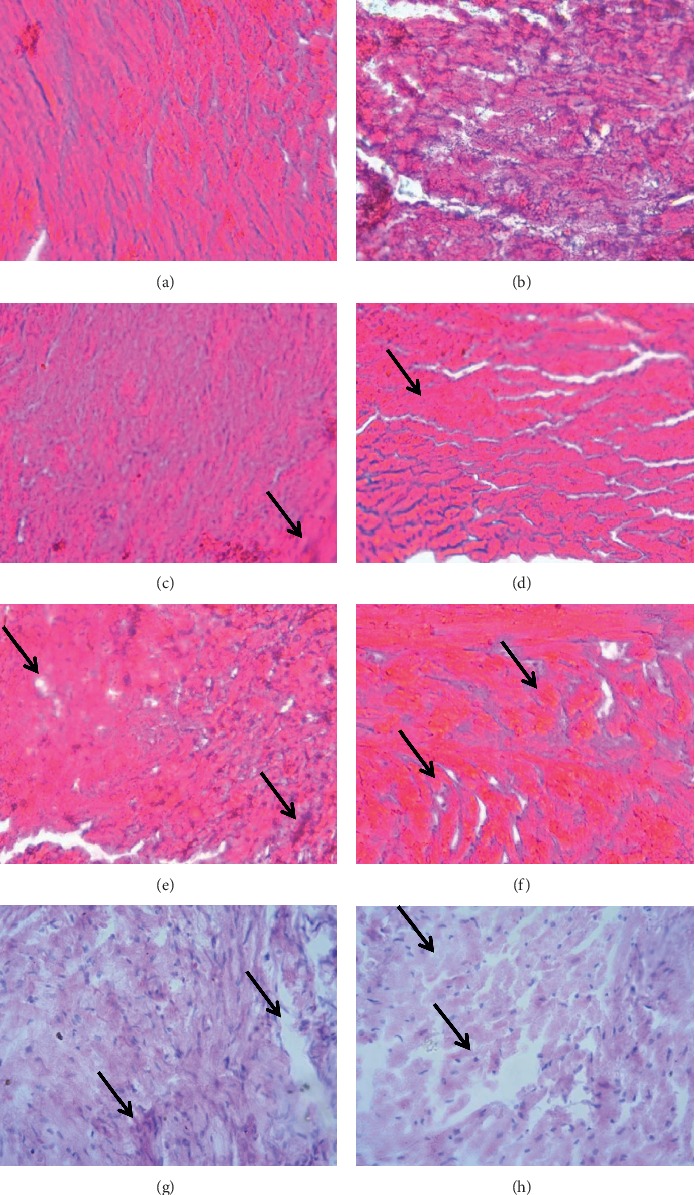
Histopathological changes in the heart tissue treated with crude extract and aqueous fraction. (a) Normal control group (normal structures of the heart). (b) Cyclophosphamide control group (congestion, degeneration of myocardial tissue, and necrosis). (c) Enalapril control group (mild necrosis). (d) Crude extract at 100 mg/kg (hypertrophy). (e) Crude extract at 200 mg/kg (edema and focal necrosis). (f) Crude extract at 400 mg/kg (edema). (g) Aqueous fraction at 100 mg/kg (edema and congestion). (h) Aqueous fraction at 200 mg/kg (edema and slight degeneration of myocardial tissue).

**Figure 6:**
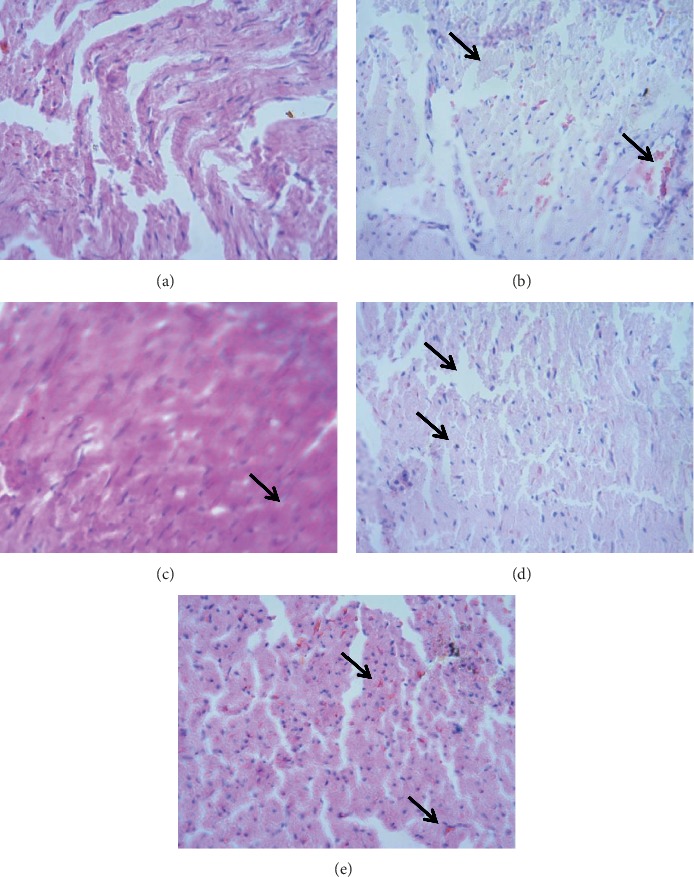
Histopathological changes in the heart tissue treated with ethyl acetate fraction. (a) Normal control group (normal structures of the heart). (b) Cyclophosphamide control group (necrosis and hemorrhage). (c) Enalapril control group (mild congestion and necrosis). (d) Ethyl acetate fraction at 100 mg/kg (nearly normal structure, edema). (e) Ethyl acetate fraction at 200 mg/kg (hemorrhage).

To determine the cardioprotective activity of *Croton macrostachyus*, a histopathologic examination was carried out in heart tissues. As shown in Figures 5 and 6, normal structures of the heart with no necrosis were observed in normal control animals. Rats treated with cyclophosphamide showed congestion, degeneration of myocardial tissue, and necrosis. Enalapril-treated groups exhibited mild diffused focal hemorrhage and necrosis. In the treatment groups with crude extract and solvent fractions of *Croton macrostachyus*, focal necrosis and hyperplasia of muscle fiber were observed, but the damage was decreased in all treatment groups (Figures 5 and 6).

## 4. Discussion

Cyclophosphamide is an alkylating agent used for cancer management [28]. It is broadly used for the treatment of leukemias, lymphomas, multiple myeloma, and rheumatic arthritis and prior to bone marrow transplantation [24]. Although it has a wide spectrum of therapeutic applications, it is a cardiotoxic drug leading to endothelial impairment and destruction of myocardiocytes [29]. One of the main reasons for cyclophosphamide-induced cardiotoxicity is oxidative stress [9]. Oxidative stress may result in endothelial dysfunction, hypertrophy, fibrosis, inflammation, apoptosis, cell migration, and angiogenesis [30].

Plants with naturally occurring antioxidant activity can be used to control homeostasis between free radicals and antioxidant stress. Medicinal plants, which are used traditionally, offer an important therapeutic effect in this regard [31].

This study set out to investigate whether *Croton macrostachyus* has cardioprotective and antioxidant effects. To evaluate the antioxidant activity of crude extract and solvent fractions, the DPPH assay method was used. The DPPH radical has an electron which is accountable for the absorbance at 517 nm and also for the observable deep purple color. During DPPH acceptance of an electron donated by an antioxidant, DPPH is decolorized to yellowish color which can be quantitatively measured from the changes in absorbance [32]. The crude extracts and solvent fractions were compared for their antioxidant activity based on their IC_50_ (concentration providing 50% inhibition).

The crude extracts and solvent fractions of *Croton macrostachyus* stem bark demonstrated free radical scavenging activity. However, standard ascorbic acid has shown strong antioxidant activity than test samples.

The phytochemical studies reported showed that stem bark of *Croton macrostachyus* constituted major secondary metabolites such as flavonoids, phenols, terpenoids, saponins, tannins, alkaloids, and steroids [19, 20]. These phytochemicals were reported to have an antioxidant activity in previous studies [33–40].

Ethyl acetate fraction exhibited strong antioxidant activity with IC_50_ of 419 *μ*g/mL relatively stronger than those of the crude extract and aqueous fractions. Ethyl acetate fraction is reported to have a high amount of total phenolics and total flavonoids [41, 42]. This could be increased due to the solubility of phenolics in ethyl acetate [43]. Thus, the highest free radical scavenging activity of the ethyl acetate fraction could be due to the presence of an increased amount of those antioxidant metabolites in higher proportions compared with the crude extract and aqueous fractions. This is similar to another finding where the ethyl acetate fraction of a different plant showed the highest radical scavenging activity [44].

In cardioprotective studies, the administration of cyclophosphamide caused a significant body weight loss compared with the normal group. This effect was consistent with previous studies [45–47]. This might be due to anorexia as a result of the damaging effect of cyclophosphamide on the gastrointestinal tract or on the appetite center (ventromedial nucleus and lateral hypothalamus) in the hypothalamus [24, 48].

Weight loss was prevented significantly by the administration of crude extract and fractions of aqueous and ethyl acetate. This may be due to the preventive ability of *Croton macrostachyus* against the damaging effect of cyclophosphamide on feeding behavior [48].

Data showed that cyclophosphamide significantly increased heart weight and heart-to-body weight ratio. These results are consistent with data presented by several researchers who worked on the cardioprotective effects of medicinal plants [26, 49]. Increment of heart weight might be due to the increased vascular hemorrhage, edema, and cardiac muscle fiber necrosis followed by the invasion of damaged tissues with inflammatory cells [24]; these effects can be confirmed by the qualitative histopathological observations elsewhere in this study. Treatment with crude extract and solvent fractions prevented the increase in heart weight and heart-to-body weight ratio. This could be attributed to the presence of flavonoids, which are reported to have a favorable effect on the heart by dilating the blood vessels resulting in reduced peripheral resistance and increased coronary circulation, delaying or preventing the progression to cardiac hypertrophy [48].

In the present study, 200 mg/kg cyclophosphamide administration has increased the levels of serum cardiac biomarkers (troponin, AST, ALT, and ALP). These results were in agreement with previous studies [24, 50]. Elevations of these enzymes in serum were associated with heart damage such as myocardial infarction, myocarditis, and heart failure [51]. This might be due to cyclophosphamide-induced direct myocardial endothelial damage and cardiomyocyte destruction or it can be due to the overproduction of reactive oxygen species accompanied by lipid peroxidation of cardiac membranes [52].

Administration of crude extract and solvent fractions of *Croton macrostachyus* decreased the release of cardiac biomarkers into the blood stream indicating amelioration of cardiac damage; this might be due to a reparative or membrane-stabilizing action of *Croton macrostachyus* [24, 26, 53]. It might also be due to the presence of antioxidants phytochemicals with cardioprotective activity like flavonoids [54, 55] and alkaloids [56]. These results were in agreement with previously reported studies [29, 48]; this observation can be mechanistically explained by the free radical scavenging activity of the *Croton macrostachyus* discussed above. The reductions in troponin, AST, and ALP levels were higher for 400 mg/kg of the crude extract compared with those of the two lower doses and the aqueous fraction. This could obviously be due to the increased levels of antioxidant phytoconstituents.

The inhibitory effect on xanthine oxidase activity might be another possible reason for the *Croton macrostachyus* protective effect. It was reported that cyclophosphamide increases myocardial xanthine oxidase activity and reduces the level of the antioxidant enzyme [9]. Xanthine oxidase is a flavoprotein, found in heart, kidney, lung, and vascular endothelium, which catalyzes the oxidation of hypoxanthine to xanthine and generates superoxide and uric acid and this leads to several diseases including CVDs [57]. In other studies, plants with antioxidant activity were also reported to have xanthine oxidase inhibitory activity [58, 59]. Phytochemical constituents like flavonoids [60], alkaloids [61], and phenolic compounds [62] were reported to inhibit xanthine oxidase enzyme.

Inflammation plays a key role in the regulation of oxidative stress and cyclophosphamide was reported to induce inflammation in cardiomyocytes [63]. Inflammation was associated with pronounced cardiac damage like heart failure [64]. The stem bark extract of *Croton macrostachyus* was reported to have an anti-inflammatory effect and this might be one possible reason for its protective effect [19].

Cyclophosphamide administration significantly increased lipid profile (TG and TC) levels. This was similar to a previously reported study [24]. Oxidative stress impairs the cell removal of free cholesterol to high-density lipoprotein, which increases the amounts of cellular free cholesterol. Generally, cyclophosphamide-induced cardiomyopathy is associated with increased cholesterol biosynthesis and esterification, reduction in cell cholesteryl ester hydrolysis, and reduced cholesterol efflux. It was reported that antioxidants have the capability to inhibit all these mechanisms [65].

Treatment with crude extract and solvent fractions of *Croton macrostachyus* decreased both TG and TC levels near to normalcy. Crude extract and ethyl acetate fraction exhibited a dose-dependent effect on both levels. This preventive effect of *Croton macrostachyus* against cyclophosphamide-induced hyperlipidemic cardiomyopathy might be due to its antioxidant activity. Previously studies have elucidated the mechanistic association of antioxidant activity with hyperlipidemic cardiomyopathy [26, 66].

Histopathological findings of heart tissues further established the results regarding the effects of *Croton macrostachyus* on anatomical (heart mass index) and biochemical parameters (Figures 5 and 6). Heart sections of normal control rats revealed normal structures of heart, while rats administered with cyclophosphamide showed congestion, degeneration of myocardial tissue, and necrosis as a confirmation of cardiotoxicity. Reversals of such damage were observed in *Croton macrostachyus* and an enalapril-treated group of animals.

## 5. Conclusion

Hydromethanolic crude extract and ethyl acetate and aqueous fractions of *Croton macrostachyus* proved to have cardioprotective activity against cyclophosphamide-induced cardiotoxicity. We recommend further study on the isolation, identification, and characterization of the active component(s) of the extracts (especially from the ethyl acetate fraction) and elucidation of their possible molecular mechanism of action.

## Figures and Tables

**Table 1:** Grouping and dosing of rats used in experiments (AQ: aqueous fraction; CE: crude extract; EA: ethyl acetate fraction).

Treatment groups	Dose
Treatment groups (CE and AQ)	
Normal control (0.9% normal saline)	0.5 mL/100 g
Cyclophosphamide (CP) control	200 mg/kg
Standard control—enalapril (EN)	10 mg/kg
Crude extract (CE 100)	100 mg/kg
Crude extract (CE 200)	200 mg/kg
Crude extract (CE 400)	400 mg/kg
Aqueous fraction (AQ 100)	100 mg/kg
Aqueous fraction (AQ 200)	200 mg/kg

Treatment groups (EA)	
Normal control (2% DMSO)	0.5 mL/100 g
Cyclophosphamide (CP) control	200 mg/kg
Standard control—enalapril (EN)	10 mg/kg
Ethyl acetate fraction (EA 100)	100 mg/kg
Ethyl acetate fraction (EA 200)	200 mg/kg

**Table 2:** Effects of crude extract and aqueous fraction on body weight, heart weight, and heart-to-body weight ratio.

Group	Dose	Relative weight changes (g)	Heart weight (g)	Heart-to-body wt. ratio (×10^−3^)
NC	NS (0.5 mL/100 g)	+6.0 ± 5.24	0.91 ± 0.01	2.77 ± 0.06

CP	200 mg/kg	−101.33 ± 7.88^*∗∗∗*^	1.17 ± 0.04^*∗∗∗*^	5.53 ± 0.17^*∗∗∗*^

EN	10 mg/kg	−65.8 ± 5.07^##^	0.89 ± 0.02^###^	3.69 ± 0.28^###^
	100 mg/kg	−68.5 ± 5.63^#^	1.01 ± 0.02^##^	3.71 ± 0.15^###^

CE	200 mg/kg	−75.0 ± 5.02	0.99 ± 0.01^##^	3.86 ± 0.19^###^
	400 mg/kg	−76.0 ± 6.22	0.88 ± 0.02^###^	3.74 ± 0.19^###^

AQ	100 mg/kg	−66.33 ± 4.07^###^	0.87 ± 0.01^###^	4.16 ± 0.11^###^
	200 mg/kg	−64.33 ± 3.40^###^	0.85 ± 0.01^###^	3.98 ± 0.11^###^

Results are expressed as mean ± SEM (*n* = 6). ^*∗∗∗*^*P* < 0.001 when compared with normal control; ^#^*P* < 0.05, ^##^*P* < 0.01 and ^###^*P* < 0.001 when compared with CP control. AQ: aqueous fraction; CP: cyclophosphamide; CE: crude extract; EN: enalapril; NC: normal control; NS: normal saline.

**Table 3:** Effects of ethyl acetate fraction on body weight, heart weight, and heart-to-body weight ratio.

Group	Dose	Relative weight changes (g)	Heart weight (g)	Heart-to-body wt. ratio (×10^−3^)
NC	2% DMSO (0.5 mL/100 g)	+8.0 ± 1.24	0.58 ± 0.04	2.16 ± 0.13

CP	200 mg/kg	−125.0 ± 1.23^*∗∗∗*^	0.81 ± 0.03^*∗∗∗*^	5.37 ± 0.23^*∗∗∗*^

EN	10 mg/kg	−93.17 ± 4.03^#^	0.54 ± 0.03^###^	3.67 ± 0.07^###^

EA	100 mg/kg	−95.83 ± 10.56^#^	0.70 ± 0.03	3.77 ± 0.17^###^
	200 mg/kg	−95.33 ± 10.66^#^	0.69 ± 0.02	3.63 ± 0.06^###^

Results are expressed as mean ± SEM (*n* = 6). ^*∗∗∗*^*P* < 0.001 when compared with normal control; ^#^*P* < 0.05 and ^###^*P* < 0.001 when compared with CP control. EA: ethyl acetate fraction; CP: cyclophosphamide; CE: crude extract; EN: enalapril; NC: normal control; DMSO: dimethyl sulfoxide.

## Data Availability

The datasets generated and/or analyzed during the current study are available from the corresponding author on reasonable request.
